# Video-Based Analysis and Reporting of Riding Behavior in Cyclocross Segments

**DOI:** 10.3390/s21227619

**Published:** 2021-11-16

**Authors:** Jelle De Bock, Steven Verstockt

**Affiliations:** IDLab, Ghent University—IMEC, Technologiepark-Zwijnaarde 122, 9052 Ghent, Belgium; steven.verstockt@ugent.be

**Keywords:** pose estimation, sports, object detection, sports data analysis

## Abstract

Video-based trajectory analysis might be rather well discussed in sports, such as soccer or basketball, but in cycling, this is far less common. In this paper, a video processing pipeline to extract riding lines in cyclocross races is presented. The pipeline consists of a stepwise analysis process to extract riding behavior from a region (i.e., the fence) in a video camera feed. In the first step, the riders are identified by an Alphapose skeleton detector and tracked with a spatiotemporally aware pose tracker. Next, each detected pose is enriched with additional meta-information, such as rider modus (e.g., sitting on the saddle or standing on the pedals) and detected team (based on the worn jerseys). Finally, a post-processor brings all the information together and proposes ride lines with meta-information for the riders in the fence. The presented methodology can provide interesting insights, such as intra-athlete ride line clustering, anomaly detection, and detailed breakdowns of riding and running durations within the segment. Such detailed rider info can be very valuable for performance analysis, storytelling, and automatic summarization.

## 1. Introduction

Over the past years, an exponential growth of cycling sensor-generated data has been observed. In contrast, the impact of these new technologies on race reporting, coaching and training, injury prevention, and safety is still limited. The main reason is that existing applications just add an additional layer of raw sensor data on top of the live broadcast (this is, for example, what Dimension Data/NTT/Velon has been doing over the past years [1,2]). This data, however, is difficult to interpret, and does not tell what is really going on and just shows, sometimes inaccurate, data and tells no real stories. Sports data science techniques are needed to improve upon this. The real goal of sports-related storytelling is to really understand what the raw sensor values mean, using a unique mix of feature engineering and machine learning. It is not about identifying the power or speed difference between Wout Van Aert and Filippo Ganna, but it is about the impact of that difference in power combined with a particular context and considering all other available data that tell the real story and help to understand the race dynamics.

In general, data about sports events are becoming more and more available. In last year’s Tour de France, for instance, NTT showcased the potential of big data in cycling by recording more than 35 million records per stage [3]. This raw data on its own is of limited practicability and relevance for fans and sports stakeholders. The real power of the generated data is uncovered when it is summarized. When this data is summarized on a per-race level, historical race and performance insights are mostly used to predict race outcome [4,5]. However, in disciplines, such as BMX, track cycling, cyclocross, or mountain biking, data can also be summarized per lap or sector within a lap. This is especially useful as these disciplines are often won or lost on a specific part of the racecourse. The digitally collected lap or sector times can help to increase the overall sports experience of fans and athletes themselves [6]. Although lap times in sport events are already very valuable and interesting, they only provide one part of global sector analysis. They are very good at illustrating who needed the least time to go from point A to point B. However, it is not only interesting to study how much time was needed, but also the path they followed and how exactly they went from point A to point B in a certain amount of time. Rather than just the raw timings, this extra meta-information might even help to understand why one athlete went faster than another. In this paper, we present a video-based approach providing an objective data-driven answer on the “why” part of the sector data analysis question. A set of tools is proposed to present riding line insights to coaches and fans. The methodology consists of a set of state-of-the-art building blocks that are brought together to offer a solution to the riding behavior task at hand.

The remainder of this paper is organized as follows: in the related work section, we discuss relevant work on sector timing and video-based ride performance analysis; under methodology, we further elaborate on the video processing pipeline and the tracked riding lines; next, the results section discusses the implementation and presents some practical results of the proposed building blocks; in the final section, we briefly summarize our main findings and contributions and point out some future work that is required to further finetune the processing pipeline.

## 2. Related Work

### 2.1. Sensor-Based Tracking and Timing

To answer the first part (i.e., the timing aspect) of the sector analysis question, several approaches have been suggested in the literature. Sector timing is often performed by some kind of wireless communication protocol. Athletes or objects wearing an active or passive tag pass through a checkpoint, which acts as a receiver or senses the tags at the checkpoint. Ultra-high-frequency RFID is one of the more popular technologies uses in sports, such as mountain biking, running, skiing, and road cycling or cyclocross. Kolaja and Ehlerova [7] performed a field study to test this technology in various sports and under various circumstances and found that this technology provides satisfactory results to accurately record checkpoint crossings. They also proposed an architecture with a backend database that (post)processes the raw data into actual checkpoint crossing timings. The big advantage of this technology is its reasonable cost, resource effectiveness, and its relative easiness to set-up [8]. Another technology that can be used for the gate crossing problem is Bluetooth low-energy technology (BLE). Sun et al. [9] studied the accuracy of this technology for gate crossing. They used a high-speed camera to quantify the accuracy of the BLE technology in different running scenarios (BLE tags worn at different locations and emitting at different signal strengths) and found that the timing error is always less than 156 milliseconds. Fasel et al. [10] implemented a gate timing hardware setup in downhill skiing based on magnetism. Magnets were placed in each of the ski gates on the downhill track and athletes wore magnetometers that ultimately provide gate timings. Timing information based on sensor information can also be very valuable for the video analysis methodology discussed in this paper. For instance, and as presented in our work, the gate-crossing and identification of riders within a segment can assist to further enhance the information extracted from the video footage (i.e., identified detected objects on the video stream).

### 2.2. Computer Vision-Based Tracking and Timing

In the previous paragraph, some available sensor-based solutions for tracking and timing were discussed, but computer vision techniques could also be used to perform performance analysis. Popular sports, such as soccer, tennis, basketball, or cycling, are usually broadcasted on national television. Although video data cannot be directly used for performance analysis, several computer vision techniques can be utilized to extract performance data for further analysis. In basketball, for instance, Arbués-Sangüesa et al. [11] proposed a methodology to extract and track the visual features of basketball players using a combination of a pre-trained pose estimation model and an additional feature extraction network. Santhosh and Kaarthick [12] performed a similar workflow but used OpenCV algorithms, such as HOG-descriptors, to detect and track players. Additionally, they also defined a homography matrix to map the detected locations on the video frames on a top-view representation of the basketball field, allowing interesting visualizations, such as heatmaps. Chakraborty and Meher [13] suggested a video-based ball detection and tracking methodology that facilitates extensive path analysis of the ball during basketball long shots. Tracking skeleton and objects is a challenging task, especially if the tracked objects are moving (e.g., jogging, walking, cycling) or brusque camera movements [14].

The fencing principle has already been extensively documented in non-sports-related literature. Chen et al. [15] defined a fence around an area that needed digital surveillance. Every time a human was detected that was entering the fence, an alert was raised. Wang et al. [16] suggested a methodology to cluster and characterize different types of trajectories of moving objects in a scene. Additionally, they also tried to semantically understand the behaviors represented by a certain trajectory (clusters). Seenouvong et al. [17] proposed a similar architecture but used a region-of-interest in which they counted vehicles with the help of computer vision algorithms.

In our ride line analysis, we will adopt a video-based approach to extract the ride lines of cyclocross riders, but as further explained in the discussion section of this paper, our approach might also benefit from additional sensor-based rider tracking. The pipeline uses a set of individual state-of-the art building blocks, which can, when brought together, perform the specific task at hand (i.e., ride line analysis). On top of these building blocks, some extensions and case-specific modifications were made.

## 3. Materials and Methods

**Figure 1:**
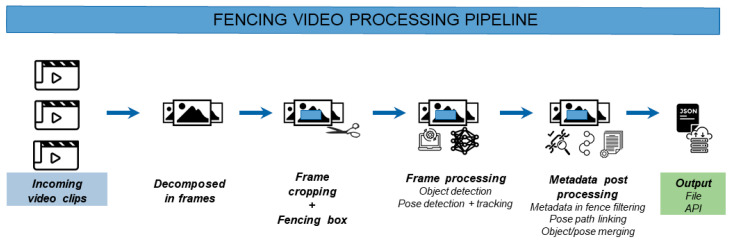
Different steps in the video processing pipeline to produce segment path data.

The proposed methodology, shown in Figure 1, consists of several consecutive steps and ultimately produces a path that bicycle riders have followed through the defined fence. In this section, we will further elaborate on each of the steps that are required to produce this path info.

**Figure 2:**
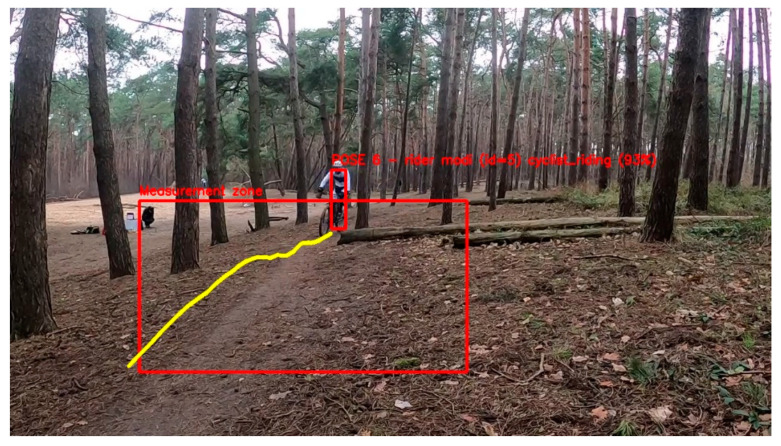
Illustration of the fence principle on a video frame of a cyclocross training session. The red rectangle is the region of interest. Results of pose detection, tracking, and post processing are illustrated by the yellow path within the fence.

The first step of the analysis consists of the decomposition and initial preprocessing of the incoming video source. The pipeline accepts both recorded video clips and live video data delivered by popular streaming formats, such as HTTP Live Streaming (HLS) and the Newtek Network Device Interface (NDI) protocols. The video data is decomposed into frames and based on the framerate of the recorded video, some frames are periodically skipped for an optimal balance between accuracy and processing time. Experiments with high-definition footage (1920 × 1080 pixels) at a framerate of 30 frames per second show that processing every third frame gives the best balance between pipeline detection accuracy and processing speed (tested on an Intel i7-10700F processor, 32 GB RAM, Nvidia RTX 2060 Super GPU). By processing every third frame, the video is processed in (near) real time. For preprocessing, the frame can be cropped to leave out irrelevant background information for further analysis (and further speed up processing times). Next, a rectangular fence is defined within the cropped region. The fence is defined as the region of the video in which movements and the behavior of riders are analyzed and is illustrated by the measurement zone rectangle in Figure 2. Only detections within this region will be considered in further analysis (see post processing subsection).

**Figure 3:**
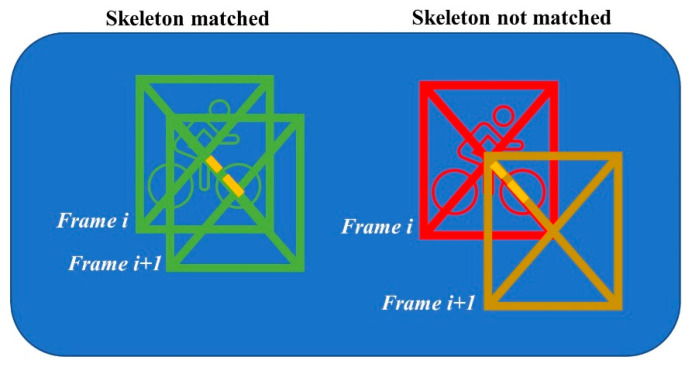
Illustration of the pose matching methodology. The yellow dotted line is the 25% threshold of the diagonal size. The poses on the left are matched between frame *i* and *i* + 1 as its distances between bounding boxes are less than 25% of the diagonal size, and the right poses are too far away to be matched.

In the following two steps, a combination of computer vision and machine learning algorithms will gather more information about the riders that are present in the fence. As a start, an Alphapose pose estimator [18] is run on the frame to detect the riders and their body keypoints. To track riders through the frames, Alphapose offers various pose tracking implementations (e.g., PoseFlow, Human ReID, or detector based). However, after thorough experimentation with the different trackers and its parameter configurations, we were not able to achieve satisfiable results. The trackers work great on pedestrians, but on skeletons that are pedaling a bicycle, the tracking often fails. Our finding that traditional tracking algorithms make considerable mistakes in a sports context is also fortified in the literature [19,20]. The tracker can fail in two different ways: first, in a tracking identifier swap, especially after partial occlusion of one skeleton behind the other. Another tracking failure occurs when a new tracking identifier is assigned to an already seen skeleton. However, for further path analysis we decided to position the camera in such a way that it films the fence from not a frontal but an overhead camera angle. This has the advantage that the fence more accurately represents the real-life coordinates and that skeleton swaps are less likely to occur as there is a better unobstructed view of the riders (e.g., riders will not be hidden after each other). With this extra prerequisite in mind, we implemented a more straightforward yet purpose tailored spatiotemporally aware tracking mechanism that mostly circumvents the mentioned shortcomings of the trackers included within Alphapose. Full details of the tracking methodology can be found in Appendix A, but we will briefly discuss the main working principles of the technique. The technique keeps track of the skeletons seen in the last five frames with its last known coordinates within the fence. When a new frame is processed, the distance matrix between the old poses’ center locations and the new pose centers is calculated. The new pose matches with an older pose if it has the minimum distance to that old pose and the distance is smaller than 25% of its diagonal size of the bounding box around the new pose (see Figure 3 for an illustration of this approach). Each time a new frame is processed, the poses older than five frames ago are also removed from the pose match dictionary. This approach works very well in cycling as cyclists travel from a starting to an end point within the fence (i.e., they never go back in the fence), so the corresponding bounding boxes are also moving similarly through the fence over time.

In cyclocross, riders ride or run with their bikes, based on the technicality and surface conditions. In the next step of the video processing pipeline, a clear distinction between each of the ridemodi a rider can adopt is made. Technical sectors might, for instance, be perfectly rideable for a rider with great technical prowess but might be completely unrideable for another less technical rider. The barriers are a great example of such a technical sector. If the barriers are relatively high and are placed at a challenging part of the course (e.g., uphill or after a corner), some less technical riders will be forced to dismount their bike and run over this course feature. Monitoring these differences among riders within the video fence can be very valuable to help understand why, how, and where riders are taking a certain line and to explain why one rider is slower than another. To answer the riding mode question, a YOLOv5 neural network was trained to detect cyclists that are either running or cycling and spectators. This knowledge is attached as metadata to the previously detected poses and its tracked pose identifiers.

**Figure 4:**
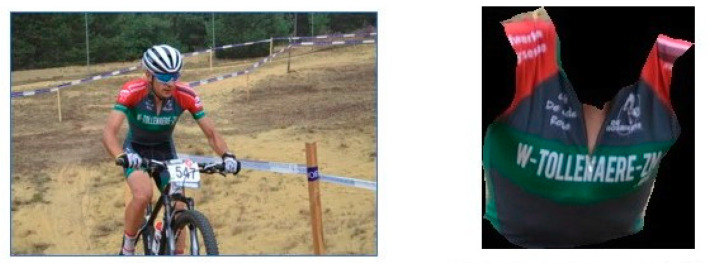
Illustration of the cropping and masking of the torso of a cyclist.

Another interesting metadata generator within the pipeline is the team (jersey) recognition module. The team jersey recognition should be capable of detecting which team a rider belongs to based on the team jerseys they are wearing. Team jerseys usually have distinct patterns with some sponsors on them. If team jerseys do not change over the years, it might be perfectly feasible to train a state-of-the-art object recognition model that has a couple of hundreds of images for each team. However, in practice, team jersey designs, sponsors, and even colors change usually every year (or sometimes even faster), which makes this approach rather unfeasible. To overcome this limitation, a methodology that only uses relatively few examples for each jersey class should be implemented. For this purpose, a transfer learning approach was used [21]. In neural network transfer learning, the trained knowledge of an existing neural network is reused to do the classification or detection task for another unseen but related problem [22]. This is usually done by removing the last output layer of the network and adding another one instead. All weights of the original network are frozen (i.e., are not trained any further), but the last layer’s weights are trained based on the (limited amount of) provided problem-specific training data. For our team classification module, we trained a RESNET18 model, with its last fully connected layer replaced by a linear layer that was retrained. In the first attempt of preparing the training data, the team jerseys were rectangularly cropped from a larger image. This introduced a lot of background noise in the image, which had a negative impact on the trained predictor’s accuracy. This shortcoming was mostly solved by an extra model that crops the relevant body parts from an image with background information [23]. For our model’s training data, we retrieved the torso from the humanparser’s generated body part segmentation output. The model is a Resnet-101 backbone adopting the Context Embedding with Edge Perceiving (CE2P) to segment the body in the different body parts. For our methodology, a network trained on the Pascal dataset to detect the following body parts: ‘Background’, ‘Head’, ‘Torso’, ‘Upper Arms’, ‘Lower Arms’, ‘Upper Legs’, and ‘Lower Legs’. For our predictor, we are only interested in the torso as these contain the logos and the distinct team patterns. The humanparser’s generated segmentation mask is used to crop out the jersey from the original image (see Figure 4). This significantly improved the model’s capability to accurately classify teams based on their jerseys. The F1 score on unseen validation data (different resolutions and view angles) increased from 68% to 78% with background subtraction and only the torso that was analyzed.

The next link in the video processing pipeline is the check of whether a rider is in the fence. This is determined by the center of the bounding box (bbox) around the joints that are detected by the Alphapose detector. If the center of the rider’s bbox is within the fence’s box, the rider is considered in the fence. 

**Figure 5:**
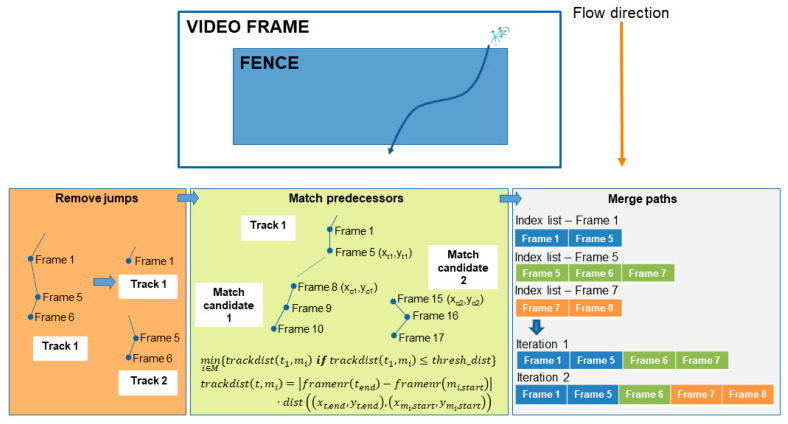
A schematic overview of the three-step pose tracking merging strategy.

After the previously mentioned pipeline elements have run, the post-processing step can now be performed. The difference with the previous steps and the post processing step is that the previous steps take place on a per frame level, but the post processing happens across multiple frames. In the real-time path analysis scenario, the post processing is initiated if the fence remains empty for 10 consecutive analyzed frames. In post-processing, the actual paths travelled by the tracked skeletons are determined. If desired, the coordinates of the paths can also be transformed into real life coordinates using a homography perspective transformation [24]. The main challenge within this post-processing step is the handling of the re-identification of the pose tracker. A pose is re-identified if the pose tracker falsely assigns a new tracking identifier to a pose that was already seen in a previous frame. The possible causes for re-identification can usually be reduced to two different categories. The first is when the Alphapose estimator fails to map the skeletons for one or more consecutive frames, which causes a jump in the subsequent positions, which is too high for our geospatial pose tracker to link it to a previous pose instance. Another culprit is when two poses are basically overlapping each other and both identifiers are mistaken for each other. With these limitations in mind, a post processing strategy can now be implemented to search and solve the re-identifications. The strategy exploits the fact that in our fenced approach, the skeletons will always travel in a consistent direction within the fence (e.g., left to right, right to left, top to bottom, or bottom to top). With this added constraint, a straightforward yet powerful geospatially aware pose path merger can be implemented. The merging process is illustrated in Figure 5 and will be briefly discussed in the next paragraph.

In summary, the merging process consists of three steps. In the first step, the paths are split based on jumps in frame numbers of the different tracks. A track is defined as a pose that was tracked over time in the fence and was assigned a tracking identifier. Tracks with non-subsequent frame sequences are split into separate tracks. This prepares the tracks for step two of the merging process, where the tracks are attempted to be merged again based on a spatiotemporal weighting function. As illustrated in Figure 5, a track has several candidates that can be matched. The selected candidate is the one with the minimum spatiotemporal distance and below a certain threshold that is set based on the fence’s dimensions. In the last step, the spatiotemporally linked tracks are iteratively matched based on the index lists of frame numbers within a track. This process stops if all paths have index lists that are non-overlapping.

The proposed pose tracking methodology combined with the merging strategy produces a set of pose tracks that can be directly used in the following steps in the video pipeline. In this next step, the pose tracks (or parts of the tracks) that are within the boundaries of the desired fence’s coordinates are extracted. To check if a skeleton of a path is within the fence, the center of the bounding box around its joints is used. Using this approach, the tracked skeletons’ coordinates stay much more consistent and less spikey as when a joint, such as the knee or foot, were used. The skeleton is considered within the fence if this center coordinate is within the fence’s bounding box coordinates. This check is performed for every frame in which the skeleton in the track was detected. A valid path in the fence is defined as a rider that is entering and exiting the defined bounding box (and has multiple detections within the fence). 

Once the valid paths have been detected, all information is available to create insightful statistics of the path a rider did follow within the fence. The extra metadata, such as the rider modi and the team jersey recognition results, are used to annotate the path with a major riding mode and the team probability scores of the rider. The combination of the video (stream) frame rate and the start frame and end frame when the rider entered or exited the fence also allows an estimate of how much time the rider did spend in that zone. In the final step, the data of the tracked skeleton across different frames within the fence is brought together and summarized, which in its turn can be published. In our video pipeline, the fence data is published to a REST API, allowing easy retrieval for other stakeholders within the cyclocross broadcasting world. As a final note, riders can be identified with a MyLaps detection loop at the start and end of the fenced zone (as will be further explained in the discussion section).

## 4. Results

In the methodology, the mechanism of the fencing framework was theoretically discussed. In this section, we will further elaborate on the implementation of the introduced building blocks and some preliminary results will be showcased.

**Figure 6:**
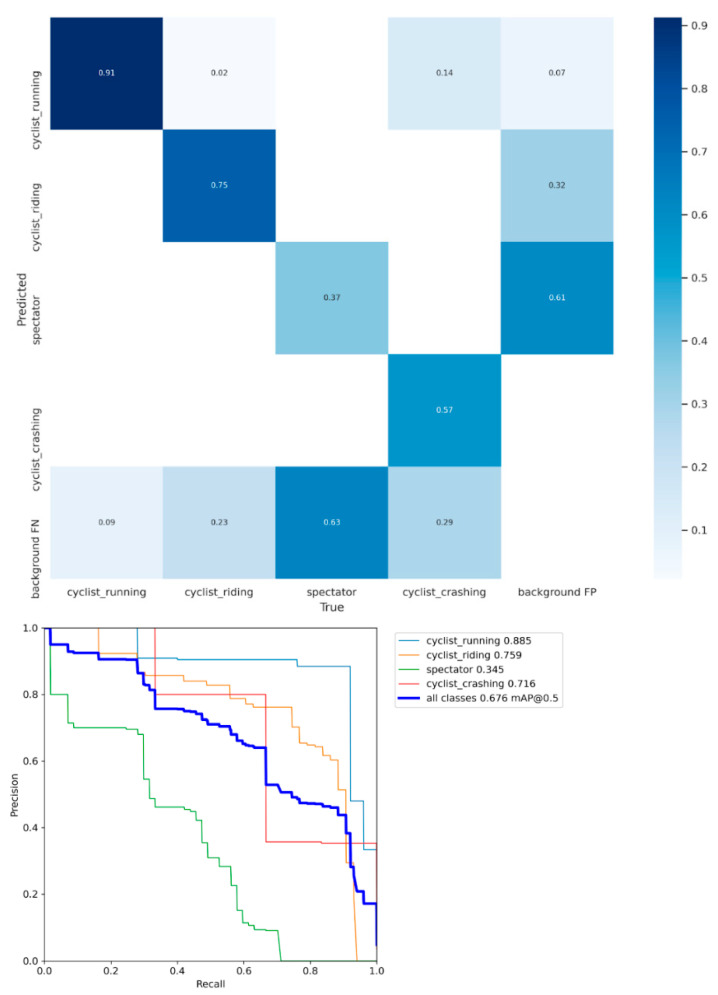
Confusion matrix and precision recall curve for the rider mode detector. As can be seen, the green line of spectators is performing significantly worse than the other classes.

**Figure 7:**
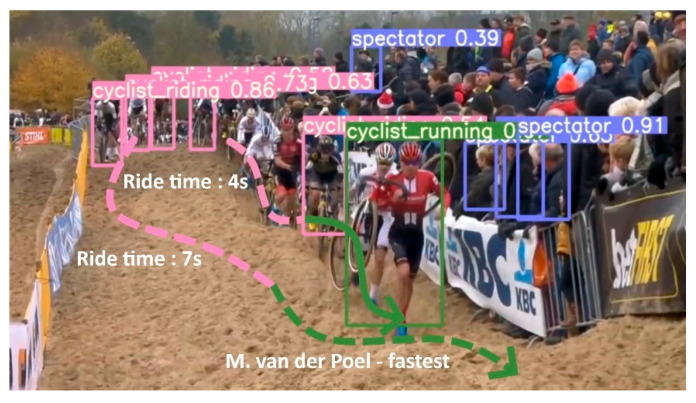
Ride mode detector run on a video extract of the World Cup race of Koksijde. The arrows show the history of the detected ride modi for that specific rider.

An important metadata generator within our pipeline is the ride mode detector. To train such a model, a training dataset of 869 images was constructed, with 747 cyclists that are riding, 457 running, 116 crashing, and 1038 spectators. The dataset was split uniformly across the categories in 75% for the training data, 20% for test, and 5% for validation. The data was used to train a YoloV5 model (yolov5s variant) for 100 epochs and achieved a mean average precision (mAP) of 68%. As can be seen in Figure 6, it is the spectator class that is degrading the model’s overall performance quite a bit as it classifies most spectators as background. This is not really a problem for this proposed methodology as we are only interested in riders and the confusion matrix shows us that there is no confusion between riders (either running or riding) and spectators. The real power of this detector is when it is used on a video sequence. In combination with the tracking results of the rider skeletons, the riders’ rider modi can be tracked over time. Figure 7 shows an illustration of the ride mode model that was run on a sequence of a men’s pro cyclocross race. The ride mode detector ran on each frame of this sequence and the color-coded arrows show the history for that rider across the video sequence. The combination of the video framerate and the fence analysis results can give an indication of the total time required to finish the sand sequence and how they did it (i.e., riding and running ratio).

**Figure 8:**
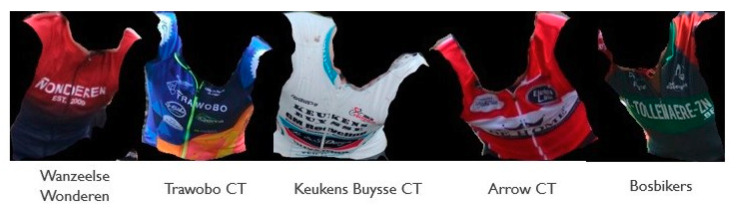
Overview of the five team jerseys that were used to train the team jersey recognition model.

**Figure 9:**
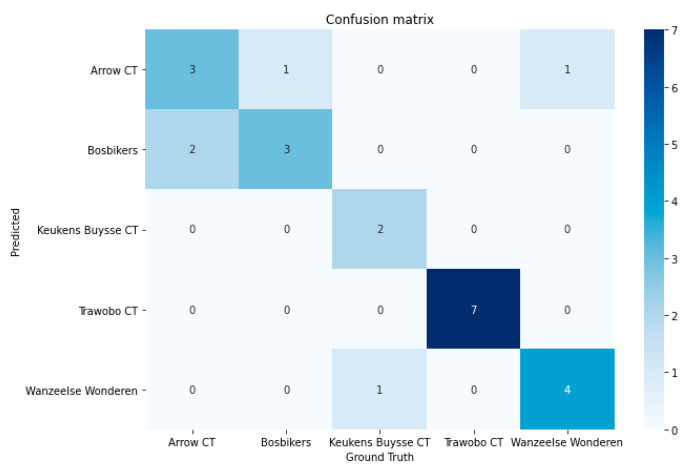
Confusion matrix of team predictions on unseen validation images trained on five team jersey classes.

The next model that was trained to serve as the metadata generator in our ride line pipeline was the team detector model. To prove the effectiveness and its relative easiness to train with few sample images, a model was trained on five teams (see Figure 8 for an overview of the corresponding jerseys). For each team, a total of eight images were used to train the final output layer of the RESNET18 model. The training images were preprocessed using a set of (random) image transformations randomly performing light condition changes, horizontal flips, slight rotations, and/or cropping. The other three images were used for testing purposes. The model achieved a 96% validation accuracy after 10 epochs of training time. The model was further validated on a number of unseen images for each team. The confusion matrix of this extra validation data is shown in Figure 9. As could be seen, some teams are still mistaken for another, but the provided shots are from multiple camera angles and zoom levels, so additional more clever cropping might still improve the model’s prediction. This experiment with the five sample teams shows the effectiveness of this approach for the specific cycling use case. As team jerseys change at least once a season, the solution needs to be easily trainable and retrainable. The experiments show that good results can be achieved with relatively few training samples. In the future work section, we will discuss other possible improvements to make the model more performant and adoptable.

**Figure 10:**
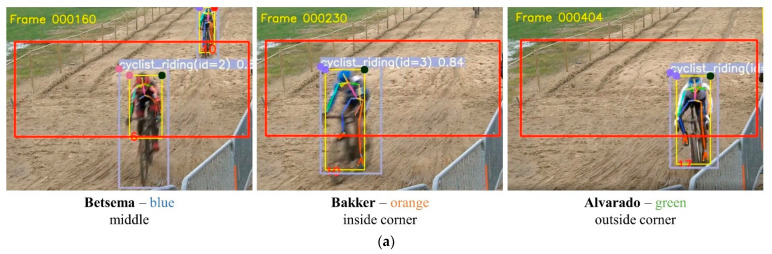

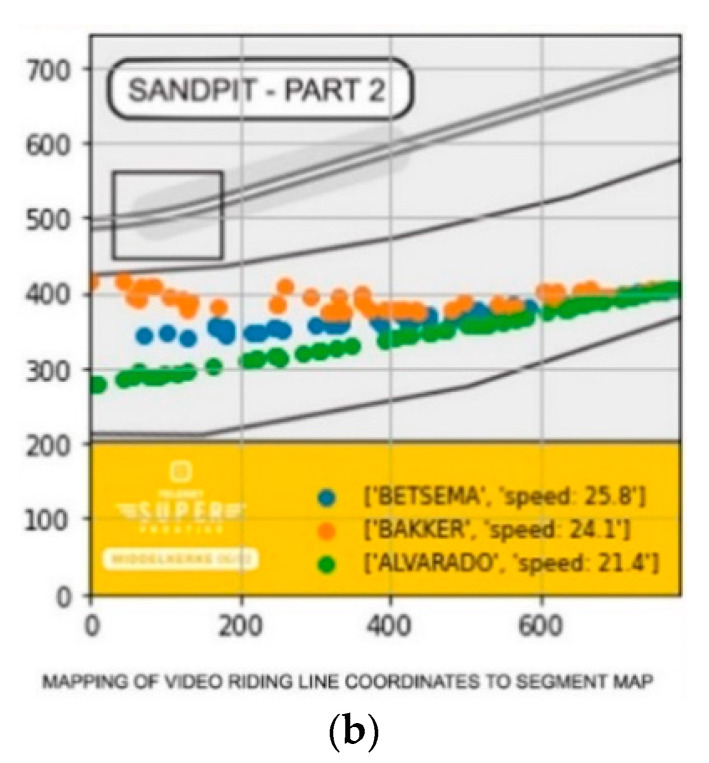
(**a**) Three female riders riding different lines through the sandpit in a professional cyclocross race. The red numbers are the tracking identifiers of the pose tracker. (**b**) Schematic overview of the produced fence paths of the various riders that went through the fence (riders go through the fence from the right to the left). Results can be linked with the riders’ pictures in the fences on (**a**).

In the previous section, we introduced the full video processing pipeline. In this paragraph, we will discuss some applications of the proposed pipeline. A first application is the direct application and analysis of the riding lines within the fence. Figure 10a,b show how this data can be visualized. As mentioned in the previous section, the coordinates of the poses were first mapped on real-life coordinates to optimally represent the true shape of the fence and paths that were followed. In the ride line graph presented in Figure 10b, we can see that the blue and orange rider tend to better follow the flow of the course than the green rider (i.e., the course curves to the right). This finding is further fortified by the screenshots of the actual video footage Figure 10a. The green rider made a technical mistake in the sand, causing a deviation from the other riders’ lines, which were more logical when the flow of the course is considered.

As a final step in the processing pipeline, the raw ride line data is also published to an API, so this information could also be used by video broadcasters to directly incorporate these near-real-time stats in the live video feed (or in race summaries or recaps afterwards). Another possibility we have been investigating is the publication of the ride line graphs presented in Figure 10b to a REST API or a Twitter account.

## 5. Discussion and Future Work

The proposed pipeline in this paper is just the starting point for further analyses and visualizations of riders’ lines and behavior on a small area on the racecourse. As the pipeline is fully modular, (metadata) pipeline elements can be added or removed as required. As an example, in sports, such as cyclocross and motocross, a MyLaps gate timing solution is often used. When the riders wear a transponder and ride over a measurement loop, their time of crossing is recorded on the MyLaps system. When such a setup is installed at the start- and endpoints of the video fences, some extra meta-information could be introduced in the video pipeline. With some post-processing and accurate time synchronization of the MyLaps and video data, rider identification and fence timing could be directly derived from the MyLaps measurements and linked to the detected skeletons in the video fence.

An extra extension of the metadata collection could be the inclusion of wearable sensor data. ANT+ is a protocol that facilitates the broadcasting and processing of sensor data, such as power, heart rate, speed, or cadence. It can be easily consumed by a USB ANT+ dongle and with one of the available Software Development Kits. The combination and time synchronization of sensor data with ride lines of the riders can provide more profound insights, such as the average power during the ridden line, maximum power, or average speed. Sensors have a distinct identifier so the pairing of a sensor with an identified rider on the video stream can be achieved.

Lastly, it is also worth mentioning that the use cases of the core detector (i.e., the skeleton detector and tracker) are not limited to cyclocross and other cycling disciplines only. A similar methodology for all other sports where riders follow a course or path can be compiled with the simple addition of sports-specific metadata generation models. As a side project, the video processing pipeline was already reused to analyze a filmed ski downhill run. The ride mode detector was changed by a ski pole and flag detector. The fencing module was also removed from the pipeline but was replaced by a pipeline post-processing element that looked at when the skier’s bounding box overlapped with the detected ski flags bounding boxes. With this information, the path the skiers took could be recreated as well as the timings of the segments between consecutive flags that were slalomed.

## 6. Conclusions

In this paper, we presented a video-based end-to-end modular and stepwise solution for detection and analysis of cyclocross riding lines. As a first step, human poses are extracted from a video frame. These poses are further identified by a ride modus and team detector. The poses from an individual frame are merged and post-processed by the pose tracker. In a final post processing stage, the ridelines are extracted from a static camera for a certain region of interest (i.e., the fence). The pipeline outputs both the raw metadata output (e.g., tracked skeletons and ride modi) and the processed rideline data. This approach was tested on a static camera positioned on the sandpit sector of a professional cyclocross race. The pipeline was able to detect and highlight irregular riding behavior. The produced insights can be used for broadcasting storytelling purposes or can be directly pushed as infographics to social networks (e.g., Twitter).

## Appendix A

1. last_id ← 0   //When a new skeleton is found it gets last id + 1 as its id
  tracked_skeletons ← []  //skeletons that were recently seen in video
2. n_frames_in_history ← 5
3. **for** frame_nr in video_frames **do**
4.    poses ← frame_resuls[frame_nr][‘poses’]
5.    pose_center_xy ← [ [x,y] **for** pose[‘coordinates’] **in** poses ]
6. pose_ids, tracked_skeletons ← map_poses(pose_center_xy, tracked_skeletons)
7.    tracked_skeletons ← cleanup_old_poses(tracked_skeletons)
8.    //Distance between tracked and new pose skeletons (omitted for readability)
9.    dist_matrix = build_distance_matrix(tracked_skeletons, pose_center_xy)
10.    used_poses ← []
11.    mapped_ids ← [null] * len(poses)
12.    **for** tracked_key **in** dist_matrix **do**
13.     min_index **← index of** minimum **of** dist_matrix[tracked_key]
14.     min_distance ← **value of** minimum of dist_matrix[tracked_key]
15.     distance_threshold ← 25% of diagonal length of the pose at min_index
16. **if** min_index **not in** used_poses **and** min_distance < distance_threshold **do**
17.    tracked_skeletons[tracked_key][‘last_seen’] = frame_nr
18.    tracked_skeletons[tracked_key][‘coordinates’] = pose_center_xy[min_index]
19.        **append** min_index **to** used_poses
20.        mapped_ids[min_index] = tracked_skeletons[tracked_key][‘idx’]
21. **end if**
22.    **end for**
23.    not_used_poses ← indices **of** poses **not in** used_poses
24. **end for**
